# Internal auditory canal metastasis mimicking a vestibular schwannoma at presentation – a case report and review of the literature

**DOI:** 10.1186/1477-7800-6-8

**Published:** 2009-03-31

**Authors:** Suat W Loo, Andrew F Dean, Philip Murray

**Affiliations:** 1 Department of Oncology, Addenbrooke's Hospital, Hills Road, Cambridge CB2 0QQ, UK; 2 Department of Histopathology, Addenbrooke's Hospital, Hills Road, Cambridge CB2 0QQ, UK; 3 Department of Oncology, Essex County Hospital, Lexden Road, Colchester, Essex CO3 3NB, UK

## Abstract

Metastasis to the internal auditory canal from breast carcinoma is extremely rare and difficult to diagnose. It radiologically mimics vestibular schwannoma and can occur as a first manifestation of systemic relapse after a long disease-free interval in patients previously treated for early breast cancer. The diagnosis is usually made retrospectively and the optimal management of such metastasis following complete resection remains undefined.

## Background

The most common lesion within the internal auditory canal is vestibular schwannoma accounting for approximately 90% of all cases in this area. Metastatic tumours are extremely rare. Here we report just such a case, radiologically mimicking a schwannoma and representing the first clinical recurrence of breast carcinoma after a 13-year interval, in order to raise awareness and highlight the difficulties in pre-operative diagnosis.

## Case presentation

**Figure 1:**
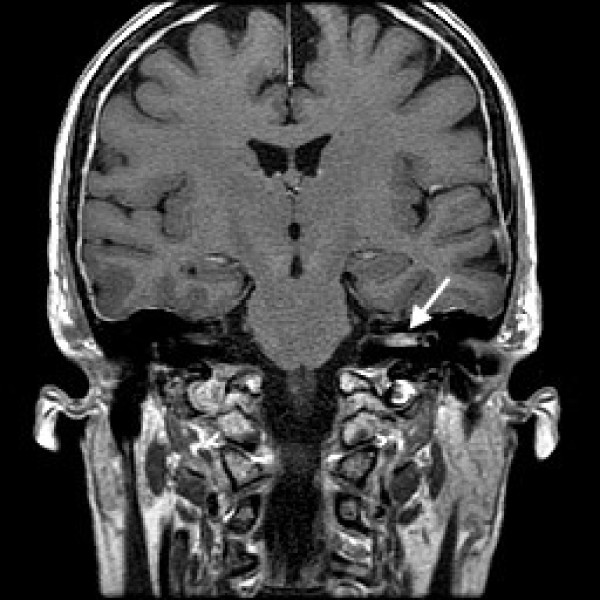
**Coronal MRI image demonstrating the intracanalicular mass within the left internal auditory canal (arrowed) which enhanced with gadolinium**.

A 66-year-old woman presented with rapidly deteriorating left-sided hearing loss, vertigo and mild intermittent dull ache in her left ear of 2 months' duration, followed by a left-sided lower motor neurone facial nerve weakness. She recalled 2 or 3 similar episodes of vertigo 2 years previously that resolved spontaneously. Thirteen years before, she had been diagnosed with invasive lobular adenocarcinoma of her right breast, treated by simple mastectomy and 5 years of adjuvant tamoxifen. 2 years later, she underwent prophylactic contralateral mastectomy. Regular follow-up had shown no evidence of disease recurrence. On present admission, physical examination revealed absent left corneal reflex and ipsilateral lower motor neurone facial nerve dysfunction (House Brackmann grade 6). The Unterberger's stepping test was to the left, Romberg was unsteady and Hitzelberger sign was positive. Pure tone audiometry showed left-sided high frequency sensorineural hearing loss. MRI revealed a 1.1 × 0.3 cm intracanalicular mass within the left internal auditory canal which enhanced with gadolinum, consistent with a schwannoma (Figure 1).

**Figure 2:**
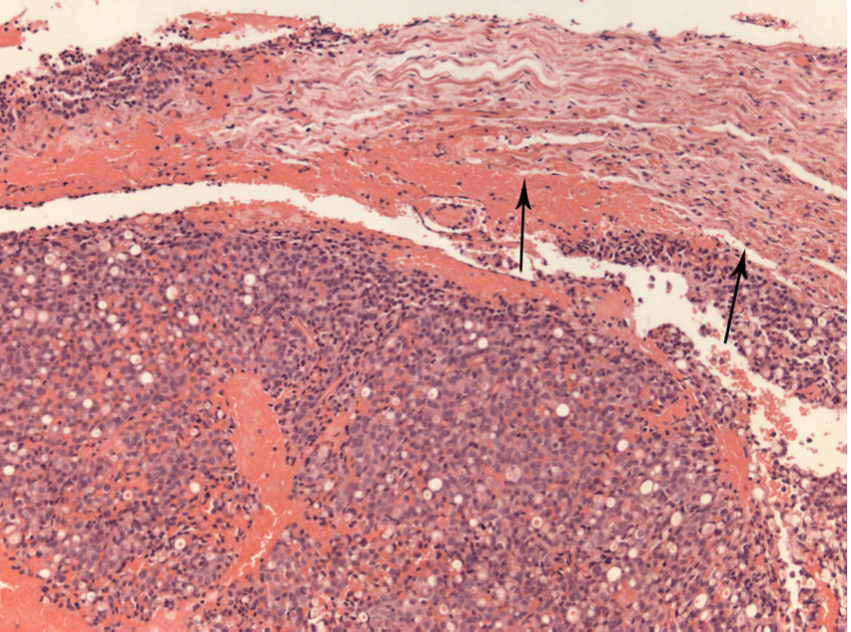
**Adenocarcinoma infiltrating nerve sheath (arrowed)**. H&E; magnification × 10.

**Figure 3:**
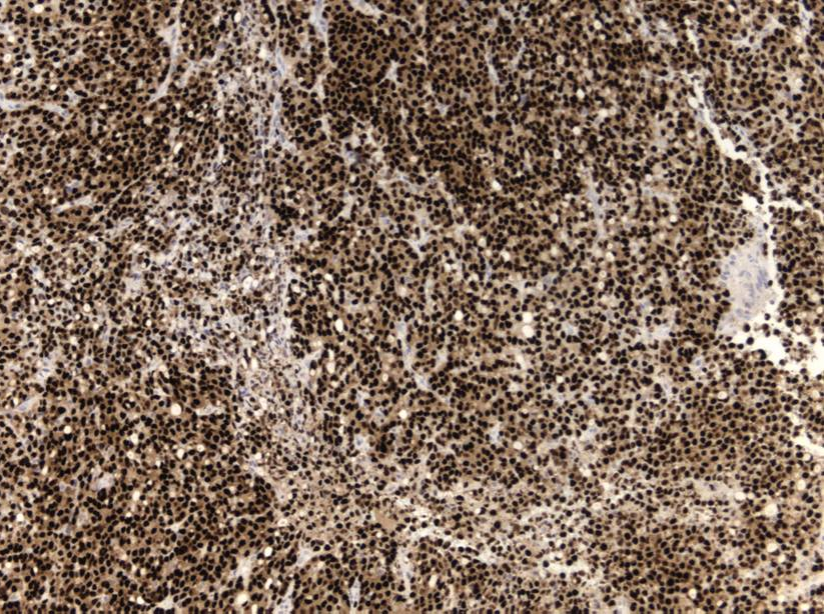
**Metastasis immunohistochemically showing oestrogen-receptor nuclear expression**. 6F11 antibody; magnification × 10.

**Figure 4:**
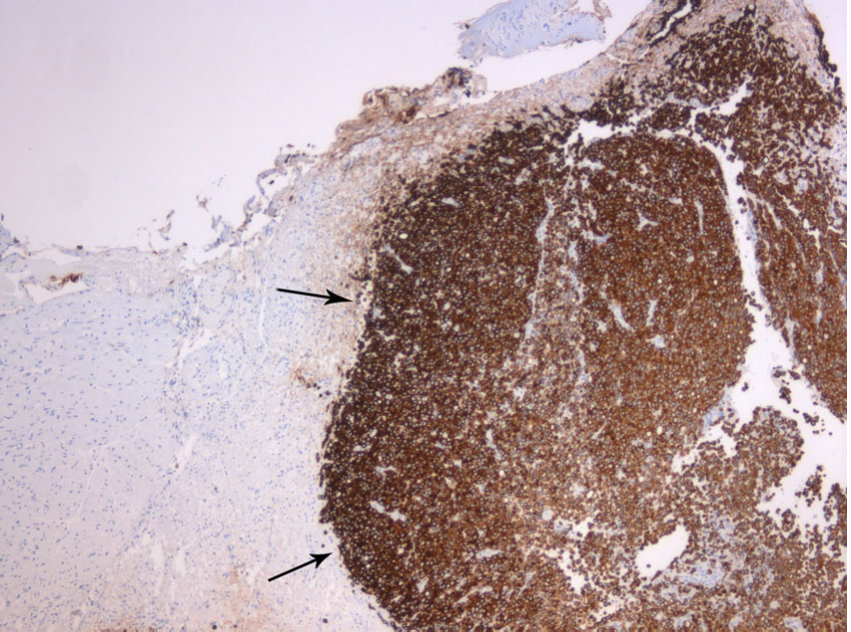
**Metastasis (arrowed) immunohistochemically showing pan-cytokeratin cytoplasmic expression**. AE1/AE3 antibody; magnification × 5.

It did not extend into the cerebellopontine cistern and the cerebellopontine angle appeared normal. The lesion was excised via a translabyrinthine approach. Intra-operatively, it resembled a vestibular schwannoma, albeit unusually vascular. Temporal bone and adjacent structures were uninvolved. Histology showed, rather than schwannoma, a metastatic adenocarcinoma infiltrating the nerve sheath that had suffered extensive axonal loss (Figure 2). No schwannoma was demonstrated. Immunohistochemistry was consistent with a breast primary with oestrogen receptor (Figure 3), pan-cytokeratin (Figure 4), cytokeratin 7, epithelial membrane antigen and E-cadherin positivity. CK20 was negative. Subsequent staging investigations revealed multiple bone metastases and she was commenced on anastrozole. There was no evidence of intracranial tumor recurrence on repeat MRI 6 months after surgery.

## Discussion

The vast majority of lesions within the internal auditory canal are benign tumors, of which vestibular schwannomas account for approximately 90%. Vestibular schwannomas are benign slow growing tumors and tinnitus is the most common initial symptom. Other common symptoms include vertigo and progressive unilateral hearing loss. Facial nerve palsy is considered a late manifestation. Metastases to the normal internal auditory canal are extremely rare. Only very few cases have ever been reported. Two of these were from breast [1,2]. Marques et al reported on a case of an isolated metastasis in the left cerebellopontine angle and internal auditory canal 16 years after treatment for a breast adenocarcinoma [1] and Guilemany et al described a patient with a pT2 pN1 M0 invasive ductal carcinoma of the breast who relapsed 5 years later with again an isolated metastasis to the left internal auditory canal and cerebellopontine angle [2]. Both of these cases were very similar to ours with a history of early breast cancer treated radically many years ago with no evidence of tumour recurrence on follow-up and presenting with symptoms of seventh and eighth cranial nerves dysfunction as the first manifestation of systemic relapse after a long disease-free interval.

Even though our MRI findings were consistent with a vestibular schwannoma, the clinical history of rapidly deteriorating sensorineural hearing loss, notably unaccompanied by tinnitus, rapidly succeeded by severe facial nerve palsy might have raised suspicion of an alternative diagnosis. Severe, unremitting pain localized to the mastoid and retromastoid areas has been noted to be a characteristic sign of metastasis to the internal auditory canal [3].

Pre-operative diagnosis of such metastasis is often difficult as there are no distinctive features on MR imaging that will help to differentiate them from the more common benign lesions. The majority of schwannomas are homogeneous, isointense in signal compared to gray matter on both T1- and T2-weighted images and enhances strongly following gadolinum. Bilateral metastases have been known to mimic neurofibromatosis type 2 [3].

Thus, the diagnosis of internal auditory canal metastasis is usually made retrospectively following surgical removal of the tumor. Immunohistochemistry is useful in identifying the tissue of origin as such metastasis can be the first sign of an undiagnosed malignancy [4,5] as well as tumor recurrence after a long disease-free period. Histology can also help to exclude the presence of a collision tumour of schwannoma and metastasis [6].

The optimal management of such metastases from a breast primary following complete resection is hard to define. In the 2 similar previous cases reported in the literature, where isolated metastasis to both the internal auditory canal and the cerebellopontine angle occurred 16 and 5 years following initial treatment for breast cancer respectively, surgical resection was noted to be complete and both went on to receive post-operative radiotherapy remaining in remission with follow-up of 3 and 18 months respectively [1,2]. Lobular carcinomas are often slower growing than ductal carcinomas and are more likely to be oestrogen-receptor positive. As our patient was shown to have widespread but asymptomatic bone metastases on further imaging which required the initiation of systemic therapy with anastrozole, local irradiation was not recommended but will be considered in the event of local relapse on subsequent scans.

## Conclusion

In conclusion, although internal auditory canal metastases are extremely rare, it should form part of the differential diagnosis when patients present with rapidly progressive and painful deficits of the seventh and eighth cranial nerves. Difficulties will still remain in distinguishing between a metastasis and a benign tumor once MR imaging reveals the presence of a lesion in the internal auditory canal.

## Competing interests

The authors declare that they have no competing interests.

## Authors' contributions

SWL, AD and PM were involved in the preparation of the manuscript. SWL performed the literature search. AD carried out the histological analysis and provided the histological slides. All authors read and approved the final manuscript.

## Consent

Written informed consent was obtained from the patient for publication of this case report and any accompanying images. A copy of the written consent is available for review by the Editor-in-Chief of this journal.
